# Contribution of Gray and White Matter Abnormalities to Cognitive Impairment in Multiple Sclerosis

**DOI:** 10.3390/ijms18010046

**Published:** 2016-12-27

**Authors:** Xiaofei Zhang, Fangfang Zhang, Dehui Huang, Lei Wu, Lin Ma, Hua Liu, Yujun Zhao, Shengyuan Yu, Jiong Shi

**Affiliations:** 1 Department of Neurology, The General Hospital of Chinese People’s Liberation Army, Fuxing Road 28, Haidian District, Beijing 100853, China; zhangxiaofei@301hospital.com.cn (X.Z.); zhangfangfang@301hospital.com.cn (F.Z.); huangdehui@301hospital.com.cn (D.H.); wulei1@301hospital.com.cn (L.W.); liuhua1978@hotmail.com (H.L.); lzhprivate@hotmail.com (Y.Z.); 2 Department of Neurology, The Affiliated Yantai Yuhuangding Hospital of Qingdao University, No. 20 East Yuhuangding Road, Yantai 264000, China; 3 Department of Radiology, The General Hospital of Chinese People’s Liberation Army, Fuxing Road 28, Haidian District, Beijing 100853, China; cjr.malin@vip.163.com; 4 Department of Neurology, Barrow Neurological Institute, St. Joseph’s Hospital and Medical Center, 240 W Thomas Rd., Ste 301, Phoenix, AZ 85013, USA

**Keywords:** multiple sclerosis, cognitive impairment, diffusion tensor imaging, voxel-based morphology

## Abstract

Patients with multiple sclerosis (MS) commonly exhibit cognitive impairments (CI). However, the neural mechanisms underlying CI remain unclear. The current study applied diffusion tensor imaging (DTI) and voxel-based morphometric (VBM) magnetic resonance imaging (MRI) techniques to evaluate differences in white matter (WM) integrity and gray matter (GM) volume between MS patients with CI and MS patients with cognitive preservation (CP). Neuropsychological assessment and MRI were obtained from 39 relapsing-remitting MS (RRMS) patients and 29 healthy controls (HCs). Patients were classified as CI or CP according to cognitive ability, and demographic characteristics and MRI images were compared. Compared with HCs, MS patients exhibited widespread damage in WM integrity, and GM loss in several regions. Compared with CP patients, CI patients exhibited more extensive WM impairments, particularly in the corpus callosum, cerebellar peduncle, corona radiata, optic radiation, superior longitudinal fasciculus, anterior limb of the internal capsule, and cingulate, as well as decreased GM volume in the bilateral caudate, left insula and right temporal lobe. MS patients with CI exhibited more significant structural abnormalities than those with CP. Widespread impairments of WM integrity and selective GM atrophy both appear to be associated with impaired cognition in RRMS.

## 1. Introduction

Multiple sclerosis (MS) is a demyelination disease affecting the central nervous system (CNS), typically characterized by motor and sensory dysfunction, and optic neuritis. Previous studies reported that 40%–70% of MS patients also exhibit cognitive impairments (CI), primarily in learning, memory, information processing speed, and executive functions [1].

CI can occur in the early stages of the disease course, even among newly diagnosed patients, and typically has severe detrimental impacts on patients’ lives [2,3]. Previous studies hypothesized that CI is associated with the extent of white matter (WM) lesion load in MS patients [4], based on the volume of T2 (transverse relaxation time) lesions visible on conventional MR imaging. However, several studies reported a weak relationship between WM damage and cognitive changes [5,6,7]. Later, several studies using diffusion tensor imaging (DTI) reported that widespread subtle pathological changes in WM microstructures (normal appearing WM; NAWM) exhibited a significant correlation with CI, whereas T2-hyperintense lesions of MS did not [8]. In addition, researchers proposed that fractional anisotropy (FA) in the uncinate fasciculus was associated with processing speed and spatial memory [9], and Hulst et al. found that WM integrity was related to cognition in CI–MS patients [10].

Extensive gray matter (GM) loss has been widely reported in patients with MS, including cortical and deep GM. Asymmetric left-sided loss of GM in the left fronto-temporal cortex and precuneus, as well as bilateral anterior cingulate gyrus and caudate nuclei atrophy, were reported in relapsing-remitting MS (RRMS) [11]. Moreover, an association between decreased GM volume and cognitive impairment was also reported. Several studies of MS patients have revealed a relationship between CI and deep GM atrophy in a range of areas, including the thalamus, hippocampus, caudate nucleus, globus pallidus, and basal ganglia, based on voxel-based morphometric (VBM) analysis, which allows quantification of GM volumes in vivo [12,13,14]. However, Hulst et al. reported that only WM integrity was significantly different between CI and cognitive preservation (CP) patients, while no difference in regional GM volume was found [10]. However, both RRMS and secondary progressive MS (SPMS) patients were enrolled in Hulst’s study [10]. Previous research indicated that cognition in SPMS patients was more frequently and severely impaired than among RRMS patients [15]. Thus, the inclusion of different disease subtypes of MS may have affected the results.

The neural mechanisms underlying CI in MS remain controversial. The discrepancy between previous results may be related to the different types of MS included in study samples. Thus, the current study aimed to further explore the neural basis of cognitive impairment by distinguishing CI and CP groups of RRMS patients according to cognitive ability, and examining them with a combination of DTI and VBM techniques.

## 2. Results

### 2.1. Multiple Sclerosis (MS) Patients vs. Healthy Controls (HCs)

#### 2.1.1. Comparison of Cognitive Test Scores

Correcting for Hamilton Anxiety (HAMA) and Hamilton Depression (HAMD) scores, MS patients exhibited worse performance in the Symbol digit modalities test (SDMT) and PASAT 3 seconds (PASAT-3″) compared with HCs. These two tests primarily reflect information processing speed and working memory (Table 1).

#### 2.1.2. White Matter Diffusion Changes—MS Patients vs. HCs

**Figure 1:**
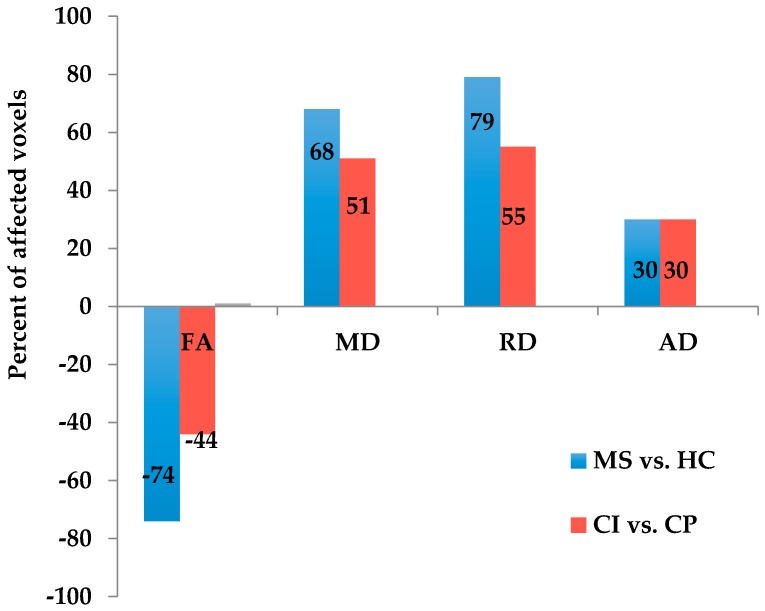
Extent of white matter (WM) integrity damage in diffusion tensor images (DTI) analyses. The bars indicate the extent of WM integrity damage. The blue and red bars indicate differences of WM diffusion between the multiple sclerosis (MS) patients and healthy control (HC) subjects, and between the cognitive impairment (CI) and cognitive preservation (CP) groups, respectively. FA, MD, RD, and AD refer to fractional anisotropy, mean diffusivity, radial diffusivity, and axial diffusivity, respectively. FA: fractional anisotropy; MD: mean diffusivity; RD: radial diffusivity; AD: axial diffusivity.

Tract-based spatial statistics (TBSS) detected significantly decreased fractional anisotropy (FA) and increased mean diffusivity (MD) and radial diffusivity (RD) in many WM tracts in MS patients compared with HCs. However, increased axial diffusivity (AD) was found to be restricted to a small number of WM tracts. Comparison of individual voxels of the WM skeleton between MS patients and HCs [10] revealed decreased FA values in 74% of the investigated WM. In addition, increased MD, RD, and AD were found in 68%, 79%, and 30% of the investigated WM, respectively, in MS patients compared with HCs (Figure 1, blue bars).

#### 2.1.3. Voxel-Based Morphometric (VBM) Comparison—MS Patients vs. HCs

**Figure 2:**
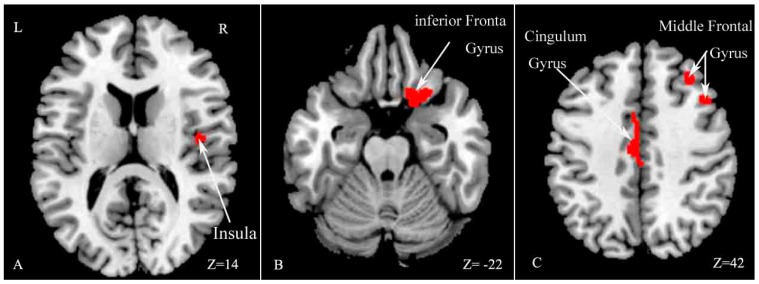
Statistical parametric mapping (SPM) regions with decreased gray matter (GM) volume in patients compared with healthy controls (*p* < 0.05, family-wise error (FWE) corrected for multiple comparisons). SPM regions exhibiting significant GM loss: right insula (**A**); right inferior frontal gyrus (**B**); right middle frontal gyrus and left cingulate gyrus (**C**). R: right; L: left.

VBM analysis revealed GM volume decreases in several regions in MS patients compared with HCs, including the right insula, middle frontal gyrus, inferior frontal gyrus and left cingulate gyrus (*p* < 0.05, family-wise error (FWE) corrected for multiple comparisons, see Table 2 and Figure 2).

### 2.2. Imaging Comparison—CI-MS Patients vs. CP-MS Patients

#### 2.2.1. White Matter Diffusion Changes—MS Patients CI vs. CP

**Figure 3:**
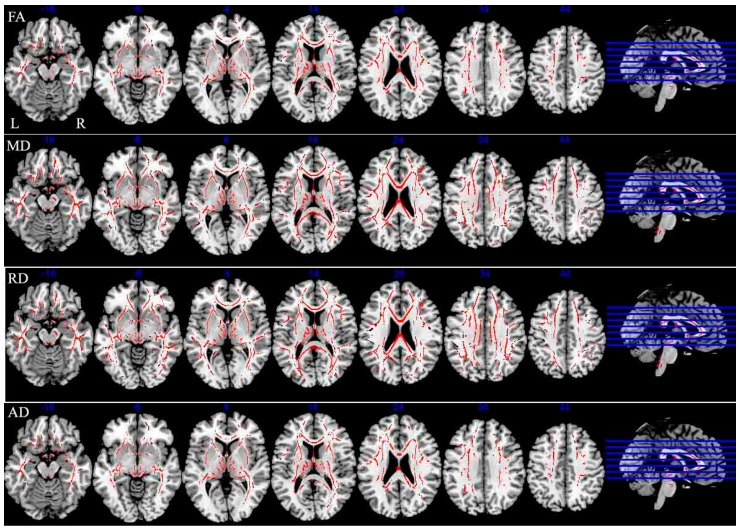
Tract-based spatial statistics (TBSS) analysis results of diffusion metrics images throughout the skeleton in CI patients compared with MS patients with CP. R: right; L: left. Red represents regions with decrease fractional anisotropy (FA), increased mean diffusivity (MD), increased radial diffusivity (RD) and increase axial diffusivity (AD) in CI patients with MS (*p* < 0.05, FWE corrected for multiple comparisons).

Decreased FA values and increased MD and RD values were observed in a large number of WM tracts across the brain in CI compared with CP patients (Figure 1). FA was decreased in 44% of the studied WM, and almost all the areas exhibiting decreased FA overlapped with areas exhibiting increased MD and RD. Statistical analyses revealed that MD and RD values were increased by 51% and 55%, respectively, in the CI group compared with the CP group (Figure 1, red bars). However, the areas showing increased AD (30% of the investigated WM) were restricted to a small number of WM tracts, including the corpus callosum, corona radiata, external capsule, superior longitudinal fasciculus, anterior limb of internal capsule, middle cerebellar peduncle, fornix (cres)/stria terminalis. In addition, these areas overlapped with regions exhibiting reduced FA, increased MD and RD (*p*_FWE correction_ < 0.05) (Figure 3 and Table 3).

#### 2.2.2. Comparisons of VBM between CI–MS Patients and CP–MS Patients

**Figure 4:**
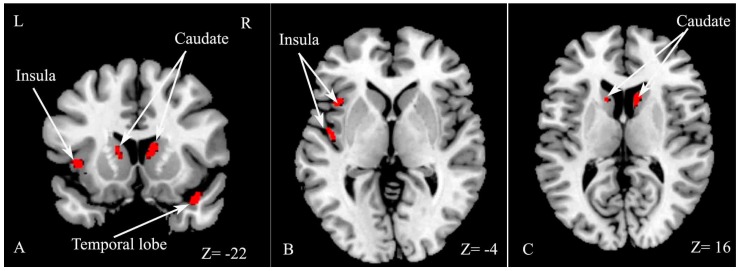
Statistical parametric mapping (SPM) regions exhibiting decreased gray matter (GM) volume in CI patients compared with CP patients (*p* < 0.05, FWE corrected for multiple comparisons). SPM regions exhibiting significant GM loss including right temporal lobe, left insula and bilateral caudate (**A**); left insula (**B**); bilateral caudate (**C**).

The VBM analysis indicated that GM volume significantly decreased in several regions, including the right temporal lobe, left insula and bilateral caudate in the CI group compared with the CP group (Table 4 and Figure 4).

## 3. Discussion

The current study revealed several main findings. First, MS patients exhibited worse performance on the SDMT and PASAT-3″ compared with HCs. We found extensive WM damage, including cortical and subcortical GM atrophy in the right insula, middle frontal gyrus, inferior frontal gyrus and left cingulate gyrus in MS patients compared with HCs. In addition, CI–MS patients exhibited worse performance on the Auditory Verbal Learning Test (AVLT), Verbal Fluency Test (VFT), PASAT-3″, Brief Visuospatial Memory Test (BVMT), and Trail making test (TMT) compared with CP–MS patients. CI–MS patients showed a widespread reduction in FA values in many WM tracts throughout the skeletons, compared with CP–MS patients. The results also revealed regional GM atrophy in several regions, including the right temporal lobe, left insula and bilateral caudate.

The current results revealed a significant difference in SDMT and PASAT-3″ performance between MS and HC, which primarily reflects information processing speed and working memory performance. This finding is consistent with previous reports [1,16]. Moreover, we found widespread WM impairment, as well as cortical and subcortical GM loss in a number of areas, including the insula, frontal gyrus and cingulate gyrus, in accord with a number of previous studies [10,11,12,17,18,19,20]. Both WM and GM abnormities are known to be involved in MS.

Further analyses revealed significant differences in verbal fluency, speed of information processing, executive function, and spatial memory between CI–MS and CP–MS patients, in accord with previous studies [10,21]. In addition, the CI group exhibited widespread WM microstructure dysfunction compared with the CP group in a range of brain regions, including the corpus callosum, cerebellar peduncle, corona radiata, optic radiation, superior longitudinal fasciculus, the anterior limb of the internal capsule, and the cingulate. The corpus callosum has previously been implicated in the speed of information processing, executive function, and verbal fluency [22]. In addition, damage to the optic radiation and superior longitudinal fasciculus have been associated with spatial memory deficits [23,24]. Hulst et al. reported a 76% decrease in FA in the WM of CI patients, and a 49% FA decrease in the WM of CP patients. On the basis of these results, the authors speculated that WM damage was related to CI–MS [10]. The current results further support the notion that WM damage is associated with cognitive dysfunction in MS.

However, no significant difference in GM volume was found between CI and CP patients in Hulst’s study, and the authors proposed that GM was not related to cognitive deficits in MS [10]. The current results revealed significant decreased GM volume, including in the right temporal lobe, left insula and bilateral caudate nucleus in CI patients compared with CP patients. The caudate nucleus has been found to be related to verbal fluency, language learning and memory [25], and the temporal lobe is implicated in memory function and information processing speed [12,24,26]. The current results indicate that atrophy of the right temporal pole, left insula and bilateral caudate nucleus may be related to CI in RRMS. The discrepancy may be related to the different disease subtypes of MS patients between the two studies.

In addition, the results revealed that CI–MS patients had a lower educational level, as well as more severe clinical disability and mental dysfunction, including anxiety and depression, compared with CP–MS patients. These findings are consistent with several previous reports suggesting that lower educational level, higher Expanded Disability Status Scale (EDSS)score and more severe mental dysfunction are related to CI [27,28,29]. A 5-year neuropsychological longitudinal study of patients with MS reported no significant change in the speed of information processing in a group of patients with more than 14 years of education, whereas patients with less than 14 years of education exhibited significant declines in information processing speed [30]. Similarly, a recent 4.5-year follow-up study indicated that increased intellectual enrichment contributed to resilience against disease-related cognitive decline [31]. In addition, compared with CP–MS patients, CI–MS patients exhibited more severe physical disabilities and abnormal moods including anxiety and depression. Previous studies reported that fatigue and abnormal mood, particularly depression, can affect patients’ cognitive function, quality of life and medical compliance [27,32]. Taken together, these findings highlight the importance of treatment for abnormal mood, particularly depression, among MS patients.

The present study involved several limitations. First, the CI patient sample size was relatively small; Second, the study did not implement a uniform set of criteria for distinguishing CI–MS patients. Most previous studies used >1.5 or 2 standard deviations (SDs) below normal data in at least two cognitive domains for identifying CI. In the current study, we used 1.5 SDs as the threshold, to enable the detection of mild CI; Third, this was a single center study. Since all patients were enrolled in the study via tertiary referrals, selection bias may have caused the sample to be non-representative of the wider population. It would be useful for future studies involving multiple centers and a larger study population to validate the current results. In addition, future studies should use more strict criteria for identifying CI (e.g., >2 SDs, or impairment in at least three cognitive domains).

It should be noted that cortical lesions were not measured in our studies and lesion-filling was not implemented either. Previous investigations have shown that lesion-filling could improve the accuracy of WM and GM volume measurements [33]. However, there is controversy about the lesion filling approach in MS. Some researchers have argued that hypointensity WM lesions in MS could be misclassified as GM in tissue segmentation that increase the measured GM volume [34,35]. In contrast, some others believe that all GM volumes except for hippocampus had smaller volumes in the original image compared to the lesion-filled image in MS patients [36]. Moreover, lesion volumes were usually identified manually, and this process might increase the subjective errors.

## 4. Materials and Methods

### 4.1. Participants

We consecutively enrolled 39 patients diagnosed with clinically definite MS with a relapsing-remitting course at the outpatient clinic of the PLAGH (The General Hospital of Chinese People’s Liberation Army) between June 2013 and December 2015. All patients were required to meet the following inclusion criteria: aged 20–55 years old, meeting the revised McDonald Diagnostic Criteria for MS [37], and no relapse within 4 weeks of testing. The exclusion criteria were as follows: metabolic or other CNS disorders with known effects on cognition, taking treatment involving steroids (prednisone > 10 mg), history of alcohol abuse or psychiatric disorders other than depression, traumatic brain injury, inability to finish neuropsychological testing because of visual acuity impairment, or MRI incompatibility (e.g., pacemaker, claustrophobia). Additionally, 29 age-, gender-, and education-matched HCs without a history of neurological diseases and with normal MRI scans were recruited. All participants underwent extensive neuropsychological, anxiety and depression evaluation and multimodal 3.0 Tesla MRI scanning. Histories pertinent to patients’ demographic and clinical characteristics, including disease duration, annualized relapse rate, and EDSS scores, were evaluated by an experienced neurologist. The study was approved by the ethics committee of the Chinese PLA General Hospital (S2013-121-01, 20 May 2013). All participants gave written informed consent. The demographic and clinical data of all subjects are summarized in Table 5.

### 4.2. Neuropsychological Evaluation and Clinical Characteristic

The neuropsychological evaluation was modified for the Chinese population based on the Minimal Assessment of Cognitive Function in Multiple Sclerosis (MACIMS) test. The modified MACIMS has been previously validated for assessing cognition in MS [16]. The battery consists of five cognitive domains commonly impaired in MS: the SDMT and PASAT (information processing speed/working memory), the BVMT-R (visual memory), the JLOT (spatial processing), the TMT (executive function), and VFT (language ability). We substituted the AVLT-Chinese Edition for the California Verbal Learning Test-Second Edition (verbal memory).

The raw score obtained from each test was converted into a standardized z-score based on the mean scores and SD of the HC participants. Patients who exhibited impairment in at least two or more cognitive domains (scoring at least 1.5 SDs below the mean test value of HCs) were classified as CI [1].

Fourteen patients (35.9%) with MS in our study were classified as CI. MS patients with CI had a lower level of education and higher EDSS, HAMA and HAMD scores than MS patients with CP, but there were no significant differences in age, onset age, disease duration, or annualized relapse rate between the two groups (Table 6).

Correcting for education level, EDSS, HAMA and HAMD scores, MS patients with CI exhibited worse performance on the Auditory Verbal Learning Test (AVLT), Verbal Fluency Test (VFT), Brief Visuospatial Memory Test (BVMT), and PASAT 3 seconds (PASAT-3″) tests, compared to the HC group, and worse performance on the AVLT, VFT, PASAT-3″, BVMT, and Trail making test (TMT) compared with CP patients. These differences were statistically significant (Table 7).

### 4.3. MRI Image Acquisition and Analysis

Brain images were obtained using a 3.0T MRI (GE 750, Waukesha, WI, USA) with a 32-channel head coil covering the whole brain. The experimental protocol included DTI images, as well as T1 and T2-weighted images.

The main sequences were performed with the following parameters:

(1) DTI sequence: echo time (TE) = minimum, repetition time (TR) = 10,000, field of view (FOV) 240 mm, matrix 128 mm × 128 mm, slice thickness 3.0 mm, *b*-value = 1000, number of slices = 44, number of directions = 25, and 0 skip between slices.
(2) Three-dimensional T1-weighted magnetization-fast spoiled gradient recalled echo (T1WI 3D-FSPGR) images: TE = min/full, flip angle 8°, slice thickness 1.2 mm, rep time = 900, matrix 256 mm × 256 mm, and 0 skip between slices. High-resolution T1-weighted images were obtained for VBM analyses.

#### 4.3.1. Diffusion Tensor Images (DTI)

DTI allowed us to quantitatively analyze the microstructural integrity of WM tracts. DTI data were preprocessed and a range of diffusion metrics, including FA, MD, RD and AD, were analyzed using the TBSS component of the FMRIB’s Diffusion Toolbox (http://fsl.fmrib.ox.ac.uk/fsl/fslwiki/). The mean FA skeleton was created with a threshold of 0.2, and the TBSS results were analyzed using the Johns Hopkins University (JHU) International Consortium for Brain Mapping (ICBM)-DTI-81 digital WM atlas (http://cmrm.med.jhmi.edu/) to identify the specific tracts exhibiting diffusion changes.

Among DTI diffusion metrics, MD reflects the diffusion velocity but not the diffusion direction, increased AD and RD reflect axonal injury and predominant demyelination respectively, and FA, as a relative index, is dependent on changes of AD and RD. Thus, decreased FA may suggest microstructural damage of WM tracts [5,17].

#### 4.3.2. VBM

The obtained T1WI 3D-FSPGR data were preprocessed and analyzed using SPM8-VBM toolboxes (http://dbm.neuro.uni-jena.de/vbm/). Voxel-by-voxel quantitative analyses of the local volume of GM between two groups were carried out using VBM.

The routine processing included transforming each participant’s T1 weighted images into the same stereotactic space, and segmenting all the spatially normalized images into GM, WM and cerebrospinal fluid (CSF). A modulation step was then performed to moderate the volume changes caused by spatial normalization. Finally, we smoothed the GM images. We then used SPM8 to perform voxel-wise statistical analysis on smoothed normalized tissue segments.

Because TBSS and VBM are spatially specific, unbiased, non-hypothesis-driven, and automated techniques for analyzing MR images, they are more easily reproducible and accurate than conventional procedures.

### 4.4. Statistical Analysis

Statistical analyses were performed using SPSS 18.0 for windows (SPSS, Chicago, IL, USA). All of the parameters were tested for a normal distribution using the Kolmogorov–Smirnov test before the statistical analysis was conducted. Independent-samples *t*-tests were used for comparison of normally distributed parameters, which were presented as means ± SD. Mann–Whitney *U*-tests were used for comparison of non-normally distributed parameters, which were presented as median values (interquartile range). The analyses of DTI and VBM data were performed using two-sample *t*-tests, treating age, sex, and education level as covariates. Differences were considered statistically significant if two-tailed *p*-values were below 0.05.

## 5. Conclusions

The current findings indicate that widespread impairment of WM integrity and selective GM atrophy may be associated with impairment of cognition in RRMS patients.

## Figures and Tables

**Table 1:** Comparison of cognition between MS patients and HC.

Cognitive Test	HC (*n* = 29)	MS Patients (*n* = 39)	*p*
AVLT-Total recall	0 ± 1.00	−0.60 ± 1.51	0.294
AVLT-Delayed recall	0 ± 1.00	−0.50 ± 1.44	0.588
VFT	0 ± 1.00	−0.31 ± 0.87	0.892
SDMT	0 ± 1.00	−0.83 ± 1.27	0.014
TMT-A	0 ± 1.00	0.78 ± 1.97	0.290
TMT-B	0 ± 1.00	0.79 ± 1.90	0.254
BVMT-R-Total recall	0 ± 1.00	−0.79 ± 1.80	0.194
BVMT-R-Delayed recall	0 ± 1.00	−0.58 ± 1.79	0.661
PASAT-3″	0 ± 1.00	−0.83 ± 1.50	0.039
JLOT	0 ± 1.00	0.06 ± 0.81	1.000

Values are mean ± standard deviation (SD); Abbreviations: AVLT = Auditory Verbal Learning Test; VFT = Verbal Fluency Test; SDMT = Symbol digit modalities test; TMT = Trail making test; BVMT-R = Brief Visuo-patial Memory Test-Revised; PASAT-3″ = Paced Auditory Serial Addition Test (3 seconds); JLOT = Judgment of Line Orientation Test; HC = healthy control.

**Table 2:** The decreased volumes of gray matter (GM) in MS patients compared to HCs (*p_FWE correction_* < 0.05).

Brain Region	Number of Voxels (mm^3^)	Peak MNI Coordinate			Brodmann	*T* Value
		*x*	*y*	*z*	Area	
Right insula	75	45	−9	12	13	5.36
Right inferior frontal gyrus	205	22	15	−24	47	5.56
Right middle frontal gyrus	268	43	18	39	9	5.68
Left cingulate gyrus	195	−7	−15	43	24	5.52

Each cluster represents the extension where local maxima are located, along with the MNI coordinates, and the *T* values of the most significant voxel are reported. A threshold of *p* < 0.05 is set for the data, and the data are corrected using the false discovery rate (FDR). MNI = Montreal Neurological Institute; FWE = family-wise error.

**Table 3:** WM diffusion changes between CI and CP groups (*p_FWE correction_* < 0.05).

White Matter Tracts	FA ↓	MD ↑	RD ↑	AD ↑
Cerebellar peduncle	Superior-R	Superior-L	Superior-Bilateral	–
	Middle	Middle	Middle	Middle
	Inferior-R	–	–	–
External capsule	Bilateral	Bilateral	Bilateral	Bilateral
Corona radiata	Bilateral	Bilateral	Bilateral	Bilateral
Corpus callosum	Genu, body and splenium	Genu, body and splenium	Genu, body and splenium	Genu, body and splenium
Optic radiation	Bilateral	Bilateral	Bilateral	–
Anterior limb of IC	Bilateral	Bilateral	Bilateral	Bilateral
Cingulate	Bilateral	Bilateral	Bilateral	–
Superior longitudinal fasciculus	Bilateral	Bilateral	Bilateral	Bilateral
Fornix (cres)/Stria terminalis	Bilateral	Bilateral	Bilateral	Bilateral

Abbreviations: CI = cognitive impairment; CP = cognitive preservation; WM = white matter; IC = internal capsule; L = Left; R = Right; ↓ indicates decreased; ↑ indicates increased.

**Table 4:** The decreased volumes of GM in CI patients compared with the CP group (*p_FWE correction_* < 0.05).

Brain Region	Number of Voxels (mm^3^)	Peak MNI Coordinate			Brodmann	*T* Value
		*x*	*y*	*z*	Area	
Right temporal lobe	117	42	18	−22	38	7.29
Left insula	70	−31	19	−4	13	7.30
Right caudate	137	12	16	10	NA	6.66
Left caudate	74	−13	10	16	NA	6.65

Each cluster represents the extension where local maxima are located, along with the MNI coordinates, and the *T* values of the most significant voxel are reported. A threshold of *p* < 0.05 is set for the data, and the data are corrected using the false discovery rate (FDR). MNI = Montreal Neurological Institute; FWE = family-wise error; NA = not applicable.

**Table 5:** Demographic and clinical characteristics in the MS group and healthy controls.

Characteristic	Healthy Control (*n* = 29)	Patients with MS (*n* = 39)	*p*
Age, year	37.79 ± 10.29	38.26 ± 9.05	0.84
Sex, F/M	17/12	23/16	0.98
Years of education	12.10 ± 4.03	11.90 ± 3.68	0.83
Disease duration, month	NA	92.33 ± 71.54	NA
Annualized relapse rate	NA	0.54 ± 0.46	NA
EDSS score	NA	2.24 ± 1.58	NA
HAMA	4.10 ± 3.19	8.33 ± 5.33	<0.01
HAMD	2.00 (0.00–3.50)	6.00 (3.00–10.00)	<0.01

Normally-distributed data are the means ± SD and analyzed by the independent-samples *t*-test. Non-normally-distributed parameters are the median (interquartile range) and analyzed by the Mann–Whitney *U*-test. Abbreviations: EDSS = Expanded Disability Status Scale; HAMA = Hamilton Anxiety Scale; HAMD = Hamilton Depression Scale; NA = not applicable; F = female; M = male.

**Table 6:** Demographic and clinical characteristics of the MS patients with CI and CP.

Characteristic	Patients with MS (CP) *n* = 25	Patients with MS (CI) *n* = 14	*p*
Age, y	37.92 ± 9.24	38.86 ± 9.02	0.76
Age at onset, y	32.08 ± 10.52	29.71 ± 8.93	0.48
Years of education	13.04 ± 2.99	9.85 ± 3.00	0.01
Disease duration (m)	80.72 ± 71.38	113.07 ± 59.50	0.18
Annualized relapse rate	0.51 ± 0.47	0.58 ± 0.45	0.66
EDSS score	1.00 (1.00–2.00)	3.50 (2.00–4.60)	<0.01
HAMA	6.40 ± 3.66	11.79 ± 6.18	<0.01
HAMD	5.12 ± 3.46	9.71 ± 4.53	<0.01

Normally distributed data are the means ± SD and analyzed by the independent-samples *t*-test. Non-normally-distributed parameters are the median (interquartile range) and analyzed by the Mann–Whitney *U*-test. Abbreviations: CP = cognitive preservation; CI = cognitive impairment; EDSS = Expanded Disability Status Scale; HAMA = Hamilton Anxiety Scale; HAMD = Hamilton Depression Scale; y = year; m = month.

**Table 7:** Comparison in cognitive tests among the MS patients with CI, MS patients with CP, and HCs.

Cognitive Test	HC (*n* = 29)	Patients with MS (CP) (*n* = 25)	Patients with MS) (CI) (*n* = 1)
AVLT-Total recall	0.00 ± 1.47	0.05 ± 1.14	−2.33± 1.74 ^a,c^
AVLT-Delayed recall	0.00 ± 1.00	0.20 ± 0.79	−1.74 ± 1.53 ^a,c^
VFT	0.00 ± 1.00	−0.02 ± 0.81	−0.85 ± 0.73 ^a,c^
SDMT	0.00 ± 1.00	−0.19 ± 0.86	−1.97 ± 1.08
TMT-A	0.00 ± 1.00	−0.18 ± 0.66	2.48 ± 2.37 ^c^
TMT-B	0.00 ± 1.00	−0.08 ± 0.65	2.29 ± 2.39 ^b^
BVMT-R-Total recall	0.00 ± 1.00	0.00 ± 1.08	−2.21 ± 1.98 ^a,c^
BVMT-R-Delayed recall	0.00 ± 1.00	0.23 ± 0.85	−2.03 ± 2.13 ^a,c^
PASAT-3″	0.00 ± 1.00	−0.02 ± 0.65	−2.29 ± 1.50 ^a,c^
JLOT	0.00 ± 1.00	0.31 ± 0.781	−0.38 ± 0.68

Values are the mean ± standard deviation (SD); ^a^ *p* < 0.05 compared with HCs; ^b^ *p* < 0.05 compared with patients with MS and CP; ^c^ *p* < 0.01 compared with patients with MS and CP. Abbreviations: AVLT = Auditory Verbal Learning Test; VFT = Verbal Fluency Test; SDMT = Symbol digit modalities test; TMT = Trail Making Test; BVMT-R = Brief Visuo-spatial Memory Test-Revised; PASAT-3″’ = Paced Auditory Serial Addition Test (3 s); JLOT = Judgment of Line Orientation Test; CI = cognitive impairment; CP = cognitive preservation; HC = healthy control.

## Abbreviations

MS	Multiple sclerosis
RRMS	Relapsing-remitting multiple sclerosis
SPMS	secondary-progressive multiple sclerosis
CI	Cognitive impairment
CP	Cognitive preservation
HC	Healthy control
AVLT	Auditory verbal learning test
VFT	Verbal fluency test
SDMT	Symbol digit modalities test
TMT	Trail making test
BVMT-R	Brief visuo-patial memory test-revised
PASAT	Paced auditory serial addition test
JLOT	Judgment of line orientation test
DTI	Diffusion tensor imaging
FA	Fractional anisotropy
MD	Mean diffusivity
RD	Radial diffusivity
AD	Rxial diffusivity
VBM	Voxel-based morphometry
WM	White matter
GM	Grey matter
MACFIMS	Minimal assessment of cognitive function in multiple sclerosis
TBSS	Tract-based spatial statistics
HAMA	Hamilton anxiety scale
HAMD	Hamilton depression scale
EDSS	Expended disability status scale

## References

[1] Liu Y., Fu Y., Schoonheim M.M., Zhang N., Fan M., Su L., Shen Y., Yan Y., Yang L., Wang Q. (2015). Structural MRI substrates of cognitive impairment in neuromyelitis optica. Neurology.

[2] Baysal Kıraç L., Ekmekçi Ö., Yüceyar N., Sağduyu Kocaman A. (2014). Assessment of early cognitive impairment in patients with clinically isolated syndromes and multiple sclerosis. Behav. Neurol..

[3] Simioni S., Ruffieux C., Bruggimann L., Annoni J.M., Schluep M. (2007). Cognition, mood and fatigue in patients in the early stage of multiple sclerosis. Swiss Med. Wkly..

[4] Benedict R.H., Zivadinov R., Carone D.A., Weinstock-Guttman B., Gaines J., Maggiore C., Sharma J., Tomassi M.A., Bakshi R. (2005). Regional lobar atrophy predicts memory impairment in multiple sclerosis. Am. J. Neuroradiol..

[5] Zivadinov R., de M.R., Nasuelli D., Bragadin L.M., Ukmar M., Pozzi-Mucelli R.S., Grop A., Cazzato G., Zorzon M. (2001). MRI techniques and cognitive impairment in the early phase of relapsing-remitting multiple sclerosis. Neuroradiology.

[6] Filippi M., Tortorella C., Rovaris M., Bozzali M., Possa F., Sormani M.P., Iannucci G., Comi G. (2000). Changes in the normal appearing brain tissue and cognitive impairment in multiple sclerosis. J. Neurol. Neurosurg. Psychiatry..

[7] Foong J., Rozewicz L., Chong W.K., Thompson A.J., Miller D.H., Ron M.A. (2000). A comparison of neuropsychological deficits in primary and secondary progressive multiple sclerosis. J. Neurol..

[8] Yu H.J., Christodoulou C., Bhise V., Greenblatt D., Patel Y., Serafin D., Maletic-Savatic M., Krupp L.B., Wagshul M.E. (2012). Multiple white matter tract abnormalities underlie cognitive impairment in RRMS. Neuroimage.

[9] Kern K.C., Gold S.M., Lee B., Montag M., Horsfall J., O’Connor M.F., Sicotte N.L. (2014). Thalamic-hippocampal-prefrontal disruption in relapsing-remitting multiple sclerosis. Neuroimage Clin..

[10] Hulst H.E., Steenwijk M.D., Versteeg A., Pouwels P.J., Vrenken H., Uitdehaag B.M., Polman C.H., Geurts J.J., Barkhof F. (2013). Cognitive impairment in MS: Impact of white matter integrity, gray matter volume, and lesions. Neurology.

[11] Prinster A., Quarantelli M., Orefice G., Lanzillo R., Brunetti A., Mollica C., Salvatore E., Morra V.B., Coppola G., Vacca G. (2006). Grey matter loss in relapsing-remitting multiple sclerosis: A voxel-based morphometry study. Neuroimage.

[12] Nocentini U., Bozzali M., Spanò B., Cercignani M., Serra L., Basile B., Mannu R., Caltagirone C., de Luca J. (2014). Exploration of the relationships between regional grey matter atrophy and cognition in mulitple sclerosis. Brain Imaging Behav..

[13] Debernard L., Melzer T.R., Alla S., Eagle J., van Stockum S., Graham C., Osborne J.R., Dalrymple-Alford J.C., Miller D.H., Mason D.F. (2015). Deep grey matter MRI abnormalities and cognitive function in relapsing-remitting multiple sclerosis. Psychiatry Res..

[14] Cavallari M., Ceccarelli A., Wang G.Y., Moscufo N., Hannoun S., Matulis C.R., Jackson J.S., Glanz B.I., Bakshi R., Neema M. (2014). Microstructural changes in the striatum and their impact on motor and neuropsychological performance in patients with multiple sclerosis. PLoS ONE..

[15] Planche V., Gibelin M., Cregut D., Pereira B., Clavelou P. (2016). Cognitive impairment in a population-based study of patients with multiple sclerosis: Differences between late relapsing-remitting, secondary progressive and primary progressive multiple sclerosis. Eur. J. Neurol..

[16] Benedict R.H., Cookfair D., Gavett R., Gunther M., Munschauer F., Garg N., Weinstock-Guttman B. (2006). Validity of the minimal assessment of cognitive function in multiple sclerosis (MACFIMS). J. Int. Neuropsychol. Soc..

[17] Sepulcre J., Masdeu J.C., Sastre-Garriga J., Goñi J., Vélez-de-Mendizábal N., Duque B., Pastor M.A., Bejarano B., Villoslada P. (2008). Mapping the brain pathways of declarative verbal memory: Evidence from white matter lesions in the living human brain. NeuroImage.

[18] Lobsien D., Ettrich B., Sotiriou K., Classen J., Then-Bergh F., Hoffmann K.T. (2014). Whole-brain diffusion tensor imaging in correlation to visual-evoked potentials in multiple sclerosis: A tract-based spatial statistics analysis. Am. J. Neuroradiol..

[19] Llufriu S., Martinez-Heras E., Fortea J., Blanco Y., Berenguer J., Gabilondo I., Ibarretxe-Bilbao N., Falcon C., Sepulveda M., Sola-Valls N. (2014). Cognitive functions in multiple sclerosis: Impact of gray matter integrity. Mult. Scler..

[20] Battaglini M., Giorgio A., Stromillo M.L., Bartolozzi M.L., Guidi L., Federico A., de S.N. (2009). Voxel-wise assessment of progression of regional brain atrophy in relapsing-remitting multiple sclerosis. J. Neurol. Sci..

[21] Chiaravalloti N.D., DeLuca J. (2008). Cognitive impairment in multiple sclerosis. Lancet Neurol..

[22] Ozturk A., Smith S.A., Gordon-Lipkin E.M., Harrison D.M., Shiee N., Pham D.L., Caffo B.S., Calabresi P.A., Reich D.S. (2010). MRI of the corpus callosum in multiple sclerosis: association with disability. Mult. Scler..

[23] Kim J.H., Park K.Y., Seo S.W., Na D.L., Chung C.S., Lee K.H., Kim G.M. (2007). Reversible verbal and visual memory deficits after left retrosplenial infarction. J. Clin. Neurol..

[24] Eichenbaum H., Lipton P.A. (2008). Towards a functional organization of the medial temporal lobe memory system: Role of the parahippocampal and medial entorhinal cortical areas. Hippocampus.

[25] Batista S., Zivadinov R., Hoogs M., Bergsland N., Heininen-Brown M., Dwyer M.G., Weinstock-Guttman B., Benedict R.H. (2012). Basal ganglia, thalamus and neocortical atrophy predicting slowed cognitive processing in multiple sclerosis. J. Neurol..

[26] Koenig K.A., Sakaie K.E., Lowe M.J., Lin J., Stone L., Bermel R.A., Beall E.B., Rao S.M., Trapp B.D., Phillips M.D. (2014). Hippocampal volume is related to cognitive decline and fornicial diffusion measures in multiple sclerosis. Magn. Reson. Imaging.

[27] DeLuca G.C., Yates R.L., Beale H., Morrow S.A. (2015). Cognitive impairment in multiple sclerosis: Clinical, radiologic and pathologic insights. Brain Pathol..

[28] Papadopoulou A., Müller-Lenke N., Naegelin Y., Kalt G., Bendfeldt K., Kuster P., Stoecklin M., Gass A., Sprenger T., Radue E.W. (2013). Contribution of cortical and white matter lesions to cognitive impairment in multiple sclerosis. Mult. Scler..

[29] Bonnet M.C., Deloire M.S., Salort E., Dousset V., Petry K.G., Brochet B., AQUISEP Study Group (2006). Evidence of cognitive compensation associated with educational level in early relapsing-remitting multiple sclerosis. J. Neurol. Sci..

[30] Benedict R.H., Morrow S.A., Weinstock-Guttman B., Cookfair D., Schretlen D.J. (2010). Cognitive reserve moderates decline in information processing speed in multiple sclerosis patients. J. Int. Neuropsychol. Soc..

[31] Sumowski J.F., Rocca M.A., Leavitt V.M., Dackovic J., Mesaros S., Drulovic J., DeLuca J., Filippi M. (2014). Brain reserve and cognitive reserve protect against cognitive decline over 4.5 years in MS. Neurology.

[32] Feinstein A. (2006). Mood disorders in multiple sclerosis and the effects on cognition. J. Neurol. Sci..

[33] Valverde S., Oliver A., Llado X. (2014). A white matter lesion-filling approach to improve brain tissue volume measurements. Neuro. Clin..

[34] Chard D.T., Jackson J.S., Miller D.H., Wheeler-Kingshott C.A. (2010). Reducing the impact of white matter lesions on automated measures of brain gray and white matter volumes. J. Mag. Reson. Imaging.

[35] Pareto D., Sastre-Garriga J., Aymerich F.X., Auger C., Tintore M., Montalban X., Rovira A. (2016). Lesion filling effect in regional brain volume estimations: A study in multiple sclerosis patients with low lesion load. Neuroradiology.

[36] Gelineau-Morel R., Tomassini V., Jenkinson M., Johansen-Berg H., Matthews P.M., Palace J. (2012). The effect of hypointense white matter lesions on automated gray matter segmentation in multiple sclerosis. Human Brain Mapp..

[37] Polman C.H., Reingold S.C., Banwell B., Clanet M., Cohen J.A., Filippi M., Fujihara K., Havrdova E., Hutchinson M., Kappos L. (2011). Diagnostic criteria for multiple sclerosis: 2010 revisions to the McDonald criteria. Ann. Neurol..

